# Understanding immune checkpoint inhibitor efficacy through spatial decoding of the lung cancer tumor immune microenvironment

**DOI:** 10.1172/JCI206316

**Published:** 2026-05-15

**Authors:** Tao Zou, John D. Minna

**Affiliations:** 1 Hamon Center for Therapeutic Oncology Research,; 2 Department of Internal Medicine,; 3 Department of Immunology,; 4 Harold C. Simmons Comprehensive Cancer Center, and; 5 Department of Pharmacology, University of Texas Southwestern Medical Center, Dallas, Texas, USA.

## Abstract

Immune checkpoint inhibitors (ICIs) have improved patient outcomes substantially in non–small cell lung cancer (NSCLC). Despite considerable effort, our understanding of the features that predict for immunotherapy response and resistance in patients remains incomplete. In this issue of the *JCI*, Isomoto and colleagues utilized a multiplex IHC platform to profile the spatial organization of the lung cancer tumor immune microenvironment, enabling the identification of spatial immune features that correlate with immunotherapy efficacy. This study enhances our knowledge of the spatial organization of features impacting ICI efficacy by identifying a three-variable spatial composite — including CD73 upregulation in *EGFR*-mutant NSCLC — that substantially outperforms PD-L1 expression in predicting immunotherapy efficacy. Moreover, it establishes spatial proteomic profiling as a platform for generating therapeutic hypotheses that are actionable and mechanistic in NSCLC.

## Spatial dissection of the NSCLC tumor immune microenvironment

Lung cancer remains the leading cause of cancer death worldwide (1). The most common form of lung cancer is non–small cell lung cancer (NSCLC), which represents approximately 80% of all new cases of this disease (1). While immune checkpoint inhibitors (ICIs) form the backbone of treatment for most cases of advanced NSCLC, the features of NSCLC tumors that govern the efficacy of these cancer immunotherapies remain incompletely defined (2).

Early studies identified PD-L1 expression and tumor mutation burden as nonoverlapping predictors of therapeutic response to PD-1/PD-L1–based ICIs in NSCLC (2). On the other hand, genomic alterations in *STK11* and *KEAP1* confer ICI resistance, particularly in the context of a concurrent *KRAS* mutation (3). Beyond immunohistochemical and genomic characterization, subsequent work has suggested that spatial organization of immune cells within the NSCLC tumor microenvironment (TME) — including the presence of stem-immunity hubs (4) and TCF1^+^ stem-like CD8^+^ T cells within tertiary lymphoid structures (TLSs) (5) — may also be associated with immunotherapy response.

**Figure 1:**
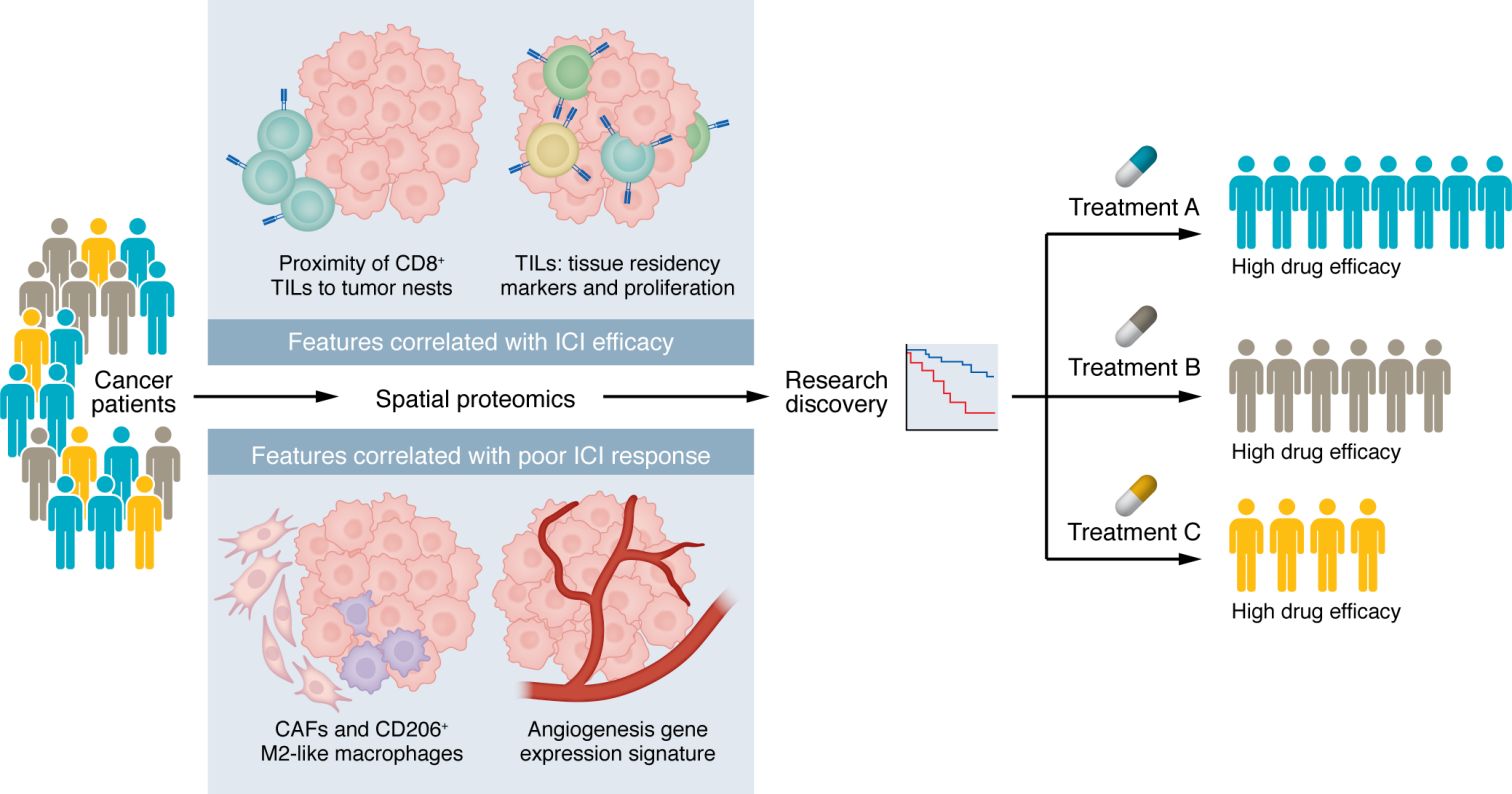
Spatial proteomics enables scientific discovery in the treatment of NSCLC. Isomoto et al. (6) identified spatial features of the NSCLC tumor immune microenvironment that are correlated with ICI efficacy (top) and ICI resistance (bottom), laying the groundwork for spatial proteomics to be utilized as a guide for scientific discovery and personalized cancer therapy.

In this issue of the *JCI*, Isomoto and colleagues used a bespoke multiplexed IHC platform combined with computational tissue segmentation to perform spatial profiling of the tumor immune microenvironment in 103 patients with metastatic NSCLC, 81 of whom received ICI therapy (6). Based on analysis of pretreatment samples, the study identified several spatial immune features correlated with immunotherapy efficacy. The proximity of CD8^+^ tumor-infiltrating lymphocytes (TILs) to tumor nests and markers of tissue residence and proliferation in TILs were associated with ICI efficacy, while CD206^+^ M2-like tumor-associated macrophages (TAMs) and fibroblast activation protein^+^ (FAP^+^) cancer-associated fibroblasts (CAFs) correlated with poorer outcomes after ICI treatment (Figure 1).

Critically, CD8^+^ TIL density in the intratumoral stromal area — the compartment typically captured by standard immunohistochemical TIL quantification and artificial intelligence–based H&E scoring — did not independently predict ICI efficacy in multivariable analysis. This finding provides a mechanistic, spatial explanation for the inconsistent clinical utility of bulk TIL assessment.

Spatial validation for Isomoto et al.’s finding is provided by a recent multiplexed immunofluorescence and deep learning study of NSCLC biopsies by Monkman et al. (7), which performed with high accuracy for predicting ICI progression-free survival >24 months. In that study, Monkman et al. used cell–cell spatial interaction features to confirm that tumor nest proximity relationships, not aggregate TIL density, constituted the critical predictive unit (7).

## Spatially localized TIL features associated with ICI efficacy

The finding that CD8^+^ TILs localized to the tumor nest predict ICI response more accurately than those in the aggregate stromal compartment raises a fundamental question: are these spatially privileged T cells recognizing tumor antigens? T cells engaged with cognate antigen should reinvigorate more robustly after ICIs than bystanders that colocalize without antigen recognition. Identifying which tumor antigens drive TIL reactivity has proved challenging because solid tumors harbor abundant bystander T cells that are reactive to common viral antigens rather than tumor-derived antigens (8, 9). Beyond classical neoantigens, cancer cells, including NSCLC cells, express noncanonical antigens: peptides derived from noncoding RNAs, UTRs, and alternative reading frames that are presented on MHC molecules and can be targeted by HLA-restricted T cells (10–12). Emerging spatial single-cell T cell receptor (TCR) sequencing technologies (13, 14) combined with evolving TCR antigen discovery platforms (15) may be used to determine whether nest-localized CD8^+^ TILs are enriched for tumor antigen reactivity and whether stromal TILs are bystanders reactive to common viral antigen.

Isomoto et al. found that TILs with markers of tissue residency within tumor nests were also associated with ICI efficacy (6), a finding supported by prior datasets in solid tumors (2, 16). While tumor-reactive TILs and bystander T cells may both express tissue residency markers, CD39 and CD103 remain imprecise surrogates (17, 18). Critically, exhausted T cells may proliferate and mediate antitumor immunity in response to ICIs, whereas tissue-resident memory (Trm) T cells may represent bystanders lacking tumor antigen reactivity; distinct ontogenies and divergent ICI responses are now well documented for these T cell subtypes (17, 18). We therefore hypothesize that CD39^+^CD103^+^ expression marks tumor-reactive tissue-resident exhausted T cells within NSCLC tumor nests that retain capacity for reinvigoration by ICIs.

Notably, expression of coinhibitory receptors, including LAG-3, TIM-3, and TIGIT, showed positive correlation with ICI response in this cohort, consistent with their role as markers of antigen-driven T cell activation in the pretreatment setting rather than terminal effector paralysis. This finding has direct implications for the interpretation of ongoing trials combining ICIs with anti–LAG-3, anti–TIM-3, or anti-TIGIT agents.

High Ki-67 positivity within Trm-like TILs likely distinguishes the TCF1^+^ progenitor-exhausted subset from TCF1^–^ terminally exhausted TILs: Ki-67^+^ and TCF1^+^ progenitor-exhausted TILs retain proliferative self-renewal capacity and can be selectively reinvigorated by PD-1/PDL-1 blockade (19), whereas the TCF1^–^ subset remains largely refractory to ICIs. These progenitor-exhausted cells are found within stem-immunity hubs and TLSs in NSCLC (4, 5), suggesting that Ki-67^+^ Trm-like TILs in tumor nests may represent effector progeny of TLS-resident precursors that have migrated upon antigen encounter.

CAFs have been reported to have diverse functions in promoting NSCLC, including mediating immunosuppression and therapy resistance (20, 21). High numbers of FAP^+^ CAFs in intratumoral stromal areas were associated with inferior ICI outcomes in the Isomoto cohort. Imaging mass cytometry and pan-cancer scRNA-seq studies have identified multiple CAF subtypes with heterogeneous survival and immunosuppressive associations (20, 21). As spatial technologies enable further CAF subtype refinement, determining which populations most potently suppress ICI efficacy in NSCLC will be essential for developing CAF-targeted therapies.

Integrating all three spatial features — high density of Ki-67^+^CD39^+^CD103^+^ Trm-like TILs in tumor nests, low CD206^+^ M2-TAM burden, and absence of FAP^+^ CAFs — identified a favorable TME (fTME) subgroup comprising 11 of 81 ICI-treated patients, whose median progression-free survival was not reached versus 5.6 months in the remainder of the cohort (HR = 0.13) (6). This effect size markedly exceeded the PD-L1–based tumor proportion score (TPS; reflecting the percentage of tumor cells that express the protein PD-L1), a standardized clinical measurement for predicting immunotherapy response. Direct comparison within this cohort also demonstrated that CD206 outperformed CD163 as a predictor of poor ICI outcome, providing marker selection guidance for future studies incorporating immunosuppressive macrophage phenotyping into NSCLC biomarker panels. The fTME composite is independently corroborated by pan-cancer analyses: a five–latent factor decomposition of ICI response across solid tumor types identified effective T cell infiltration and TGF-β microenvironment activity as universal axes of ICI resistance (22), and the LORIS composite scoring framework demonstrated stable ICI outcome prediction across 2,881 patients from 18 tumor types (23).

## Insights into ICI therapy in EGFR-mutant lung adenocarcinoma

ICI therapy is currently not recommended for patients with *EGFR*-mutant lung adenocarcinoma. Isomoto et al.’s finding that *NT5E*-encoded CD73 (ecto-5′-nucleotidase) protein expression was highly enriched in *EGFR*-mutant NSCLC — versus *EGFR* WT — thus identifies both a mechanism for ICI resistance in *EGFR*-mutant lung adenocarcinoma and a therapeutic target of importance. The proposed mechanistic circuit is as follows: oncogenic EGFR activation drives autophagy and extracellular ATP release, which CD73 hydrolyzes to immunosuppressive adenosine, in turn promoting M2-TAM accumulation, aberrant angiogenesis, and TGF-β upregulation that collectively suppress TIL function. This circuit nominates CD73 blockade, which is currently under clinical investigation in multiple solid tumor types (24), as a potentially synergistic combination partner with ICI in *EGFR*-mutant NSCLC, complementary to the VEGF inhibition strategy (25).

Transcriptomic profiling in Isomoto et al. (6) revealed enrichment of angiogenesis gene expression signatures in *EGFR*-mutant NSCLC, consistent with recognition of CD206^+^ M2-TAMs and FAP^+^ CAFs as VEGF sources in the TME. The HARMONi-A trial demonstrated that ivonescimab — a bispecific antibody cotargeting PD-1 and VEGF — significantly improved progression-free survival versus chemotherapy alone in previously treated *EGFR*-mutant NSCLC (26), validating VEGF inhibition as a strategy for rendering the *EGFR*-mutant TME more responsive to ICI. These findings collectively suggest that modulating the functional outputs of immunosuppressive TME populations, rather than depleting them directly, may represent a tractable approach in oncogene-driven NSCLC.

## Implications and future directions

In summary, Isomoto and colleagues present a landmark spatial proteomic characterization of the NSCLC tumor immune microenvironment. We view this platform as a research-grade proof of concept: its discriminatory power is compelling, but prospective multi-institutional validation, reagent and computational pipeline standardization, and reduction to a deployable clinical surrogate are required before routine implementation. The dual mechanistic circuit in the *EGFR*-mutant TME — linking EGFR to CD73 and to excess adenosine, and also linking EGFR to VEGF — opens complementary immunotherapeutic strategies, with CD73 blockade as a rational ICI partner that is potentially synergistic with the VEGF inhibition approach validated by HARMONi-A.

Looking forward, we envision this spatial proteomic platform as the foundation of a precision medicine workflow for individual patients with NSCLC. Integration with oncogenotype, RNA-seq, scRNA-seq, and epigenomic data will reveal whether specific tumor molecular states drive favorable or unfavorable TME configurations and whether oncogene-targeted therapies can beneficially shift them (Figure 1). When spatial profiling reveals an unfavorable TME, the identified suppressive mechanisms, exemplified here by CD73-driven adenosine and VEGF-mediated immunosuppression, serve as mechanistic road maps for individualized combination immunotherapy design. Extension to the tumor cell surfaceome will enable rational antibody-drug conjugate and bispecific T cell engager deployment. Moreover, as an extension of the TCR and antigen discovery framework discussed above, patient-specific neoantigen and T cell clone identification will support peripheral blood monitoring, neoantigen vaccination, and CAR T development for patients with NSCLC.

## Conflict of interest

JDM receives licensing royalties from the NIH and the University of Texas Southwestern for distribution of human tumor cell lines. TZ’s spouse has equity interest in Novartis.

## Funding support

This work is the result of NIH funding, in whole or in part, and is subject to the NIH Public Access Policy. Through acceptance of this federal funding, the NIH has been given a right to make the work publicly available in PubMed Central.

- National Cancer Institute K08 CA262169 (to TZ).
- Cancer Prevention and Research Institute of Texas grant RR250063 (to TZ).
- Lung Cancer SPORE P50 CA070907 (to JDM).
- Simmons Comprehensive Cancer Center grant P30 CA142543.

## Version 1 05/15/2026

Electronic publication
